# New Drugs and Preparations

**Published:** 1894-11-03

**Authors:** 


### NEW DRUGS AND PREPARATIONS.

[We shall be glad to receive, at our Office, 428, Strand, London, W.C., from the manufacturers, specimens of all new preparations and drugs which may be brought out from time to time.]

### HYDRASTIS CANADENSIS IN SWEATING.

Olzweski (*Wien. Med. Blätter*, April, 1893) speaks favourably of the antihydrotic action of Hydrastis canadensis. He gave it in 93 cases altogether where sweating was a prominent symptom. Of these 73 cases were pulmonary phthisis, and the results were remarkably good. A dose of 20 to 30 minims of the liquid extract generally proved sufficient, but if not, the same amount given two or three times daily had the desired effect. The sweating frequently stopped at once, and on recommencing was checked by a similar dosage. In some cases vomiting occurred, but otherwise no unpleasant effects were ever noted.

### DULCIN.

In the *Therapeut. Monatshefte* of February, 1894, Aldehoff describes the action of a sweet body recently introduced under the name dulcin or sucrol. It is about 200 times as sweet as sugar, and has been recommended as a substitute for it. Its chemical name is para-phenetolcarbamide. It differs from saccharin in having a purely sweet taste, without any trace of after-bitterness, and it has been described as without action on the organism, but Aldehoff has found it to have considerable activity, which is undoubtedly a disadvantage as compared with saccharin. Dogs which got 15 grains per diem by the mouth began after a few days to suffer from vomiting, loss of appetite, apathy, and emaciation. The urine became dark, sometimes intensely brownish-red, but did not affect the spectrum; it contained bile, and almost simultaneously the animals showed more or less icterus, although the stools kept their normal colour. Death occurred in about three weeks, the jaundice gradually becoming more intense. Post-mortem the various bile channels were always found perfectly patent. This action is the more remarkable when it is borne in mind that dulcin is closely allied chemically to phenacetin. The author has not investigated the matter more fully as yet, his object in publishing this preliminary notice being to enjoin caution in the use of even small doses of dulcin.

### ACETIC ETHER AS A STIMULANT.

After investigating the effects of acetic ether on the circulation and respiration of rabbits, Krautwig (*Cbl. f. Klin. Med.*, April, 1893) strongly recommends its trial as a stimulant in man, beginning with a dose of 8 minims subcutaneously, to be increased if necessary. He found it especially valuable in stimulating respiratory effort after it had been artificially diminished by morphine. On the other hand, he found that ordinary ether diminished the respiratory capacity under similar circumstances, and is of opinion that it still further deadens the half-paralysed centres, this being true especially of large doses. The effects of acetic ether were found to be very lasting. Moderate doses had no depressing effect on the heart and blood-pressure. It is still to be seen whether equally good results will be obtained in men.

### TOLYPYRINE.

This is antipyrine (phenyldimethyl-pyrazolon) in which a hydrogen atom of the phenyl group has been replaced by the radical methyl ($CH_3$). Its chemical name is para-tolydimethyl-pyrazolon. It occurs in colourless crystals with a very bitter taste, soluble in about ten parts of water, and easily soluble in alcohol. Like antipyrine, its watery solution gives an intensely red colour on the addition of ferric chloride, and green with nitrous acid. It is eliminated in the urine.

Guttmann[1] has tested its antipyretic value in six cas of enteric fever, five of pneumonia, two of facial erysipelas, two of scarlet fever, one of septicæmia, one of otitis media, and one of gangrene of the scrotum. Given in daily doses of 60 grains, 15 grains each time with intervals of an hour, he found that it lowered the temperature about 1 deg. to 3½ deg. Fahr.; the fall commencing within an hour after administration, reaching its lowest point in five or six hours, and then slowly rising again. Therefore, if the drug be commenced about midday, the temperature will be maintained nearly at the normal until next morning. There is some perspiration, and occasionally vomiting, but no other disagreeable symptoms were ever noticed. It is in every way as satisfactory as antipyrine, and 60 grains of it have an effect equal to about 75 to 90 grains of the latter.

As an antirheumatic it proved also highly satisfactory; 60 grains in 24 hours in mild cases of acute articular rheumatism reduced all the symptoms in one or two days; in more severe cases its effects were not so marked. In neuralgia and headache it also acted well. The author concludes that it is as useful as antipyrine, and being cheaper might advantageously replace it in practice.

[1] Berlin klin. Wochenschr., 1893, p. 249.

